# Nanosized and metastable molybdenum oxides as negative electrode materials for durable high-energy aqueous Li-ion batteries

**DOI:** 10.1073/pnas.2024969118

**Published:** 2021-11-23

**Authors:** Jeongsik Yun, Ryota Sagehashi, Yoshihiko Sato, Takuya Masuda, Satoshi Hoshino, Hongahally Basappa Rajendra, Kazuki Okuno, Akihisa Hosoe, Aliaksandr S. Bandarenka, Naoaki Yabuuchi

**Affiliations:** ^a^Physics of Energy Conversion and Storage, Physik-Department, Technische Universität München, 85748 Garching, Germany;; ^b^E-Conversion 80799 Munich, Germany;; ^c^Department of Applied Chemistry, Tokyo Denki University, Tokyo 120-8551, Japan;; ^d^Department of Chemistry and Life Science, Yokohama National University, Kanagawa 240-8501, Japan;; ^e^Research Center for Advanced Measurement and Characterization, National Institute for Materials Science, Ibaraki 305-0044, Japan;; ^f^Energy and Electronics Materials R&D Laboratories, Sumitomo Electric Industries, Ltd., 554-0024 Osaka, Japan;; ^g^Advanced Chemical Energy Research Center, Yokohama National University, Kanagawa 240-8501, Japan;; ^h^Elements Strategy Initiative for Catalysts and Batteries, Kyoto University, Kyoto 615-8245, Japan

**Keywords:** aqueous battery, metastable, rock-salt oxide

## Abstract

The development of inherently safe energy devices is a key challenge, and aqueous Li-ion batteries draw large attention for this purpose. Due to the narrow electrochemical stable potential window of aqueous electrolytes, the energy density and the selection of negative electrode materials are significantly limited. For achieving durable and high-energy aqueous Li-ion batteries, the development of negative electrode materials exhibiting a large capacity and low potential without triggering decomposition of water is crucial. Herein, a type of a negative electrode material (i.e., Li*_x_*Nb_2/7_Mo_3/7_O_2_) is proposed for high-energy aqueous Li-ion batteries. Li*_x_*Nb_2/7_Mo_3/7_O_2_ delivers a large capacity of ∼170 mA ⋅ h ⋅ g^−1^ with a low operating potential range of 1.9 to 2.8 versus Li/Li^+^ in 21 m lithium bis(trifluoromethanesulfonyl)amide (LiTFSA) aqueous electrolyte. A full cell consisting of Li_1.05_Mn_1.95_O_4_/Li_9/7_Nb_2/7_Mo_3/7_O_2_ presents high energy density of 107 W ⋅ h ⋅ kg^−1^ as the maximum value in 21 m LiTFSA aqueous electrolyte, and 73% in capacity retention is achieved after 2,000 cycles. Furthermore, hard X-ray photoelectron spectroscopy study reveals that a protective surface layer is formed at the surface of the negative electrode, by which the high-energy and durable aqueous batteries are realized with Li*_x_*Nb_2/7_Mo_3/7_O_2_. This work combines a high capacity with a safe negative electrode material through delivering the Mo-based oxide with unique nanosized and metastable characters.

## Significance

This study describes a high-energy and durable aqueous battery system with metastable and nanosized Mo-based oxides used as high-capacity negative electrodes. A wider electrochemical window is achieved with concentrated aqueous electrolytes through which highly reversible Li storage without the decomposition of water molecules is achieved for the Mo-based oxides. A full cell with an Mn-based oxide shows good capacity retention over 2,000 cycles. X-ray absorption spectroscopy reveals that the solid-state redox reaction of Mo ions reversibly proceeds in aqueous electrolytes for the metastable Mo oxide. This study opens a way to develop high-energy, durable, and safe batteries on the basis of metastable and nanosized oxides with aqueous electrolyte solutions.

The demand for better rechargeable batteries is continuously increasing due to the penetration of electric vehicles (EVs) into the automotive market and the installation of stationary energy storage systems (ESSs) for renewable energy provision. For energy storage applications, Li-ion batteries (LIBs) are the best option thanks to their high energy density and efficiency (1). For instance, LIBs already power most of the state-of-the-art portable devices, and current EVs are equipped with 50 kWh of battery power to drive over 300 km on a single charge. Moreover, grid-scale ESSs should be operated at the megawatt-hour to gigawatt-hour level (2, 3). However, flammable organic electrolytes currently used in LIBs raise safety concerns upon scaling up, and battery explosion hazards are, therefore, a severe problem, especially for large-scale applications. The safety issues can be fundamentally addressed by utilizing aqueous electrolytes, which possess several beneficial characteristics such as higher ionic conductivity, environmental benignancy, and high safety (4).

The first aqueous LIB was reported by Dahn et al. using LiMn_2_O_4_ and VO_2_ (B) as a positive and negative electrode, respectively (5). However, the major demerit of aqueous Li-ion batteries is found in low operating voltage, which results in lower energy density compared to LIBs using organic electrolytes. This problem intrinsically originates from the thermodynamic limitation (i.e., the narrow electrochemical stability window of aqueous electrolytes [the operating voltage window is typically <1.8 V, considering sluggish kinetics of water electrolysis]) (6, 7). Many positive electrode materials of conventional LIBs can be operated in aqueous electrolytes because their relevant redox potentials are located within this stability window (4, 8). In contrast, the choice of negative electrode materials is limited, and the hydrogen evolution reaction cannot be easily avoided at the surfaces of conventional negative electrode materials (e.g., graphite used for commercial LIBs). Various negative electrode materials were reported to date, such as LiV_3_O_8_, TiP_2_O_7_, LiTi_2_(PO_4_)_3_, polyaniline, polyimide, MoO_3_ coated with polypyrrole, and poly(naphthalene four formyl ethylenediamine). Nevertheless, they demonstrated specific energy density lower than 100 Wh ⋅ kg^−1^ and very poor cycling stabilities (7, 9–15).

In 2015, a new era of high-voltage aqueous LIBs had begun with the introduction of the so-called “water-in-salt” electrolytes by Suo et al. (16). A much wider stability window, ∼3 V, is realized in the water-in-salt electrolyte [i.e., saturated 21 m lithium bis(trifluoromethanesulfonyl)amide (LiTFSA) aqueous electrolyte (equivalent to 5.2 M aqueous solution)] (17). Such electrolytes are also called concentrated electrolytes and hydrated melts (18). A significant reduction of the water concentration effectively suppresses the oxygen evolution reaction, leading to higher decomposition potential upon oxidation (17). Hydrogen evolution potential on reduction is also shifted toward more negative potential due to the presence of protective solid electrolyte interphase layers, which are formed by its sacrificial decomposition of bis(trifluoromethanesulfonyl)amid (TFSA^–^) anions (16). It was reported that a full cell of LiMn_2_O_4_/Mo_6_S_8_ in a 21 m LiFTSA aqueous electrolyte shows an energy density of 84 Wh ⋅ kg^−1^ at a 0.2-C rate (16). In the following year, a 2.5-V aqueous LIB cell consisting of LiMn_2_O_4_ and carbon-coated TiO_2_ was demonstrated using the so-called “water-in-bisalt” electrolyte and exhibited high energy density of 100 Wh ⋅ kg^−1^ (19). The available energy density of aqueous LIBs was further extended to 130 Wh ⋅ kg^−1^ by using Li_4_Ti_5_O_12_ as a negative electrode material, which exhibits the operating voltage of 1.55 V versus Li metal (theoretical capacity: 175 mA ⋅ h ⋅ g^−1^) (18, 20, 21). Nevertheless, the high energy density was achieved only at higher rates because of the unavoidable and simultaneous decomposition reaction of water molecules upon electrochemical cycles to high voltages. Moreover, a high rate of charge/discharge limits the utilization of the negative electrode capacity, which is estimated to be ∼100 mA ⋅ h ⋅ g^−1^.

**Fig. 1.**
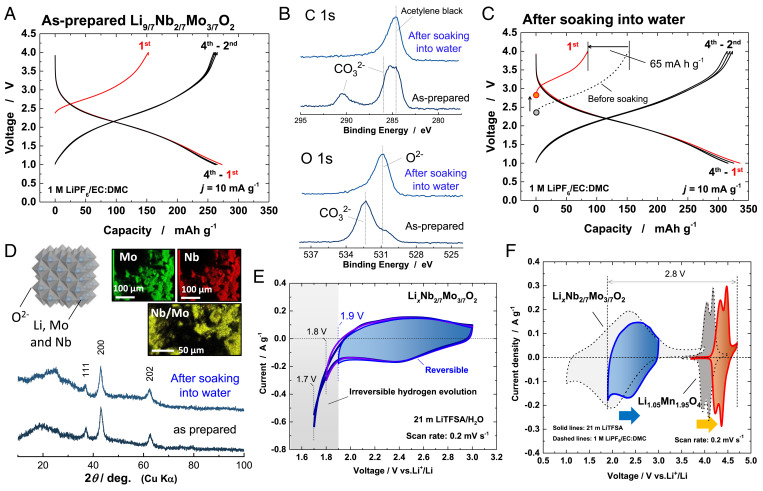
Characterization of Li*_x_*Nb_2/7_Mo_3/7_O_2_. (*A*) Charge/discharge curves (in a nonaqueous cell) of as-prepared Li_9/7_Nb_2/7_Mo_3/7_O_2_. (*B*) SOXPES spectra of C 1s and O 1s core levels of the sample before and after soaking in water. (*C*) Charge/discharge curves of Li*_x_*Nb_2/7_Mo_3/7_O_2_ after soaking in water. (*D*) X-ray diffraction (XRD) patterns of the sample before and after soaking in water and energy-dispersive X-ray spectroscopy (EDX) elemental maps of the sample after soaking in water. A schematic illustration of the crystal structure of Li*_x_*Nb_2/7_Mo_3/7_O_2_ drawn using the program VESTA (33) is also shown. (*E*) Cyclic voltammograms of Li*_x_*Nb_2/7_Mo_3/7_O_2_ in 21 m LiTFSA at a scan rate of 0.2 mV ⋅ s^−1^. A blue vertical line shows the lowest potential limit available in 21 m LiTFSA aqueous electrolyte. (*F*) Cyclic voltammograms of Li_1.05_Mn_1.95_O_4_ and Li*_x_*Nb_2/7_Mo_3/7_O_2_ in 21 m LiTFSA (solid lines) and 1 M LiPF_6_/EC:DMC (dashed lines), respectively.

In this study, a class of negative electrode materials exhibiting high capacity and high durability (i.e., a metastable and nanosize molybdenum oxide with a rock-salt structure) is proposed for aqueous LIBs. Our group originally designed an Li-excess molybdenum oxide containing niobium ions, Li_9/7_Nb_2/7_Mo_3/7_O_2_, as a high-capacity positive electrode material for nonaqueous batteries (22). The nanosized molybdenum oxides were prepared by mechanically milling the mixture of LiMoO_2_ and Li_3_NbO_4_. Mechanical milling is an effective approach to synthesize metastable materials by applying mechanical energy (23, 24). As prepared, Li_9/7_Nb_2/7_Mo_3/7_O_2_ delivers a discharge capacity of ∼275 mA ⋅ h ⋅ g^−1^ in 1 M LiPF_6_/EC:DMC (3:7) in an Li half-cell as displayed in Fig. 1*A*. During the discharge, the voltage starts to gradually decrease from 3.0 to 1.0 V on the basis of the Mo^3+^/Mo^6+^ three-electron redox reaction with the average operating voltage of 1.9 V versus Li metal, which is too low to be used for positive electrode materials. However, this electrode material can be potentially utilized for high-capacity negative electrode materials for aqueous LIBs owing to the adequate operating voltage. Note that the initial charge capacity of the as-prepared sample is smaller than that of the initial charge capacity, indicating that the sample is partially oxidized. Because of the low open-circuit voltage of the sample (2.37 V versus Li), the sample is easily oxidized by contacting with a trace of moisture (22). This process results in the formation of defect sites (Li_9/7–_*_y_*Nb_2/7_Mo_3/7_O_2_) in the bulk and LiOH at the surface of oxide particles, coupled with hydrogen generation. The surface of Li_9/7–_*_y_*Nb_2/7_Mo_3/7_O_2_ before and after soaking in water was further analyzed by soft X-ray photoelectron spectroscopy (SOXPES). Surface analysis clearly reveals the presence of Li_2_CO_3_ as shown in Fig. 1*B* and *SI Appendix*, Fig. S1*A*, indicating that surface LiOH absorbs gaseous CO_2_. Herein, the sample was soaked in water to remove surface Li_2_CO_3_ and to further oxidize the molybdenum oxides, which is beneficial to utilize the sample as negative electrode material. After the soaking of the as-prepared sample in pure water, the initial charge (oxidation) capacity was reduced by 65 mA ⋅ h ⋅ g^−1^ in an Li half-cell and a large discharge capacity of 330 mA ⋅ h ⋅ g^−1^ was obtained as shown in Fig. 1*C*. The smaller initial charge capacity originates from that further oxidation of the sample by water and elimination of Li_2_CO_3_ and LiOH from the surface of oxide particles. Indeed, after soaking in pure water, the pH value of the solution is changed to >12, and after drying the solution in air, white powder was obtained, which is assigned to a mixture of Li_2_CO_3_ and LiOH (*SI Appendix*, Fig. S1*B*). Surface Li_2_CO_3_ is dissolved in water, and the cleaner surface for oxide particles is evidenced as shown in Fig. 1*B*. Also, the oxidation of Mo ions after soaking in water is also supported by X-ray absorption spectroscopy (XAS) (*SI Appendix*, Fig. S1*C*). However, no significant change in crystal structure after the water soaking is observed, as shown in Fig. 1*D*. A cation-disordered rock-salt structure is retained after the soaking with the lattice parameters’ change from 4.206 to 4.205 Å. Energy-dispersive X-ray elemental mapping confirms that Nb and Mo ions are homogenously distributed (Fig. 1*D*). These observations indicate that Li ions were topotactically extracted from the oxide. Furthermore, the surface impurity formed by the oxidation was washed away into water, increasing the reversible capacity (see Fig. 1*C*).

In order to assess the electrode performance and a stable operating potential limit, the Li*_x_*Nb_2/7_Mo_3/7_O_2_ electrodes were examined with cyclic voltammetry (CV) in a 21 m LiTFSA aqueous electrolyte. The potential ranges were set as from 3.0 to 1.9, 1.8, and 1.7 V versus Li/Li^+^ at a scan rate of 0.2 mV ⋅ s^−1^. It should be noted here that the electrochemical measurements in aqueous electrolytes were performed using an Ag/AgCl reference electrode, but the respective electrode potential is referred to as Li/Li^+^ in the figures instead of Ag/AgCl for the sake of ease for comparing the different systems. The reversible reaction proceeds in the voltage range of 3.0 to 1.9 V in the aqueous electrolyte as can be seen in Fig. 1*D*. However, an irreversible hydrogen evolution reaction occurs when the voltage is set to below 1.9 V. Fig. 1*E* compares the CV curves of Li*_x_*Nb_2/7_Mo_3/7_O_2_ in the 21 m LiTFSA aqueous electrolyte (solid lines) and 1 M LiPF_6_ in EC:DMC = 3:7 (dashed lines). The data of Li_1.05_Mn_1.95_O_4_ (positive electrode) is also displayed. By utilizing the 21 m LiTFSA electrolyte, reversible CV curves for both positive and negative electrodes are obtained similarly to those in the organic electrolyte. Notably, the redox potentials of the lithiation and delithiation of both electrodes in the aqueous electrolyte are positively shifted by ∼0.3 V due to the Nernstian shift because of high Li-ion concentration (18).

**Fig. 2.**
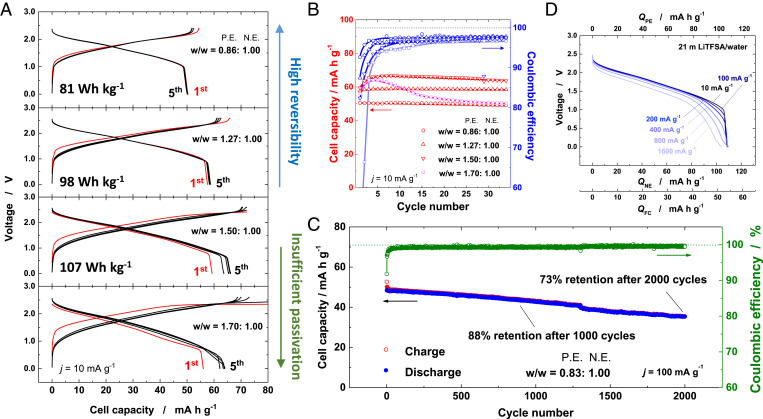
Electrochemical properties of Li_1.05_Mn_1.95_O_4_/Li*_x_*Nb_2/7_Mo_3/7_O_2_ full cells. (*A*) Comparison of charge/discharge curves of Li_1.05_Mn_1.95_O_4_/Li_x_Nb_2/7_Mo_3/7_O_2_ full cells consisting of different weight ratios of positive electrodes to negative electrodes at a rate of 10 mA ⋅ g^−1^ and (*B*) their capacity retention and Coulombic efficiency for 35 cycles in 21 m LiTFSA/H_2_O. (*C*) Long-term cycling stability performance of the full cell for 2,000 cycles at a rate of 100 mA ⋅ g^−1^ and (*D*) rate capability of the full cell in 21 m LiTFSA/H_2_O.

From the data shown in Figs. 1*E* and 2, V-class aqueous batteries are possibly fabricated by combining both electrodes, Li*_x_*Nb_2/7_Mo_3/7_O_2_ and Li_1.05_Mn_1.95_O_4_, as negative and positive electrode materials, respectively. Full cells were assembled with different mass loading ratios for the positive/negative electrodes in order to balance the different specific capacity of Li*_x_*Nb_2/7_Mo_3/7_O_2_ and Li_1.05_Mn_1.95_O_4_. The mass loading ratios of the positive electrodes were varied with respect to the negative electrode from 0.86 to 1.7. The assembled cells were cycled at a slow rate of 10 mA ⋅ g^−1^. The respective galvanostatic charge/discharge curves in the 21 m LiTFSA aqueous electrolytes are compared in Fig. 2*A*, and corresponding capacity retentions of the cells are also shown in Fig. 2*B*. When the mass loading ratio of the positive electrode to negative electrode is limited to 0.86, excellent reversibility and a small initial irreversible capacity are noted within a charge/discharge voltage range of 2.4 to 0 V. The reversible capacities based on positive/negative electrode materials are respectively provided in *SI Appendix*, Fig. S2. The cell delivers a capacity of ∼50 mA ⋅ h ⋅ g^−1^ on the basis of the total sum of the active material weight of both positive/negative electrode materials, and the average discharge voltage is calculated to be 1.62 V. The energy density of the cell reaches ∼81 Wh ⋅ kg^−1^, which is comparable to that of LiMn_2_O_4_/Mo_6_S_8_ in the 21 m LiTFSA aqueous electrolyte (16). Note that such excellent reversibility was achieved in the so-called water-in-salt electrolyte and that the cell cannot be charged to 2 V in 1 M LiTFSA aqueous electrolyte (*SI Appendix*, Fig. S3**)**. Coulombic efficiency for the initial cycle reaches 93% at a rate of 10 mA ⋅ g^−1^, and 97.5% efficiency was retained after 10 cycles (see blue open circles) in Fig. 2*B*. These values are comparable to those of the data in the nonaqueous electrolyte (*SI Appendix*, Fig. S4).

As the mass loading of the positive electrode increases, energy density of the Li cells is enhanced accordingly, indicating that larger reversible capacities are available for the negative electrode. When the mass loading of the positive electrode is increased to 1.27, enhanced cell capacity and energy density (∼100 Wh ⋅ kg^−1^) are obtained, and the charge cutoff voltage is also shifted up to 2.6 V. Moreover, good capacity retention was achieved with the average discharge voltage of 1.7 V. Further increase in energy density of ∼107 Wh ⋅ kg^−1^ was realized at the cell with the mass ratio of 1.50:1.00 for the positive/negative electrode, respectively. An observed reversible capacity of the negative electrode reaches ∼170 mA ⋅ h ⋅ g^−1^ (*SI Appendix*, Fig. S5). This value is the highest reversible capacity as negative electrode materials for aqueous LIBs as summarized in Table 1. The initial Coulombic efficiency is reduced to 82%, but it gradually increases to 96% during the initial several cycles (see blue inverted triangles in Fig. 2*B*). It is also noted that the average discharge voltage is slightly decreased to 1.6 V. Coulombic efficiency of the negative electrode is lowered, which is associated with unavoidable hydrogen generation as increase in the mass ratio. Nevertheless, gradual passivation occurs at the surface of the negative electrode, leading to an increase in Coulombic efficiency on cycles. In general, such imbalanced Coulombic efficiency for positive/negative electrodes is a crucial problem to design full cells, leading to the loss of reversible capacity. However, nanosized Mo oxides were not completely delithiated after soaking in water (Fig. 1*A*), and the issue of the imbalanced Coulombic efficiency is effectively solved as illustrated in *SI Appendix*, Fig. S6. Good cyclability in Fig. 2*B* indicates that the surface of the negative electrode is effectively passivated in the initial several cycles even though the average voltage is slightly decreased in the passivation process. Further increase in the mass ratio of the positive electrode to 1.70 facilitates even higher capacity utilization for the negative electrode (>170 mA ⋅ h ⋅ g^−1^) as shown in *SI Appendix*, Fig. S7. Nonetheless, large irreversible charge/discharge capacities are seen at the initial several cycles, and relatively faster capacity fading is observed as shown in Fig. 2*B* (open pink squares). This fact indicates that passivation is insufficient, and therefore capacity degradation on cycles is unavoidable.

**Table 1.** Comparison of specific discharge capacities of negative electrode materials, electrolyte, plateau voltage, and their energy densities of aqueous Li-ion full cells

Negative/positive electrodes	Specific capacity of negative electrodes (mAh ⋅ g^-1^)	Electrolyte	Average voltage (V)	Energy density (Wh/kg)	Current density (mA ⋅ g^-1^)	Ref.
LiTi_2_(PO_4_)_3_/LiFePO_4_	110	1 M Li_2_SO_4_	0.9	50	100	34
LiTi_2_(PO_4_)_3_LiMn_2_O_4_	138	1 M Li_2_SO_4_	1.5	60	1,000	6
Li_4_Ti_5_O_12_/LiCoO_2_	102	Li(TFSI)_0.7_(BETI)_0.3_·2H_2_O	2.35	130	1,300	18
Mo_6_S_8_/LiMn_2_O_4_	128	21 m LiTFSI	1.5∼2.0	84	7	16
TiO_2_/LiMn_2_O_4_	150	21 m LiTFSI + 7 m LiOTf	2.1	100	75	19
VO_2_/LiVOPO_4_	146	20 m LiTFSI	1.4	84	100	35
c-TiO_2_/LiMn_2_O_4_	116	32 m KOAc + 8 m LiOAc	2.2	84	22	36
Polyimide/LiCoO_2_	160	5 M LiNO_3_	1.10	80	100	14
Li*_x_*Nb_2/7_Mo_3/7_O_2_/LiMn_2_O_4_	170	21 m LiTFSA	1.7	107	10	This study

Not many publications are found related to aqueous LIBs cycled at slow rates because of unavoidable side reactions of water decomposition.

In order to study the long-term cyclability, the full cell was cycled at an accelerated rate. The full cell shows excellent cycling stability over 2,000 cycles at a rate of 100 mA ⋅ g^−1^, and the final discharge capacity after 2,000 cycles is recorded as 73% of the initial capacity as seen in Fig. 2*C*. Capacity retention with the aqueous electrolyte is better than that of the full cell with the nonaqueous electrolyte (*SI Appendix*, Fig. S8). The high cycling stability of the full cell might be associated with the suppressed dissolution of electrode materials into the electrolyte due to the lack of free solvents (25–27) and the presence of the protective solid electrolyte interphase (SEI) layers. Surface layer formation is not anticipated for the organic electrolyte in this voltage range. Rate capability of the Li cells is compared in the aqueous and organic electrolytes. Fig. 2*D* and *SI Appendix*, Fig. S9 compare rate capability of the full cells at different current densities in 21 m LiTFSA/water and 1 M LiPF_6_/EC:DMC, respectively. In the given potential range, the cells exhibit an excellent rate performance even at a very fast charge rate (1600 mA ⋅ g^−1^) in both electrolytes. In the aqueous electrolyte, the discharge voltage is dropped to a small extent at the accelerated discharge rates, and the rate performance is inferior compared to that in the organic electrolyte. A Ragone plot of the full cells is also shown in *SI Appendix*, Fig. S10. Inferior rate capability for the aqueous electrolyte could be attributed to the slightly lower ionic conductivity of 21 m LiTFSA/water (0.90 S/m) to that of 1 M LiPF_6_/EC:DMC (1.2 S/m) (16). The surface layer also would contribute to the increase in polarization on electrochemical cycles. The measured impedance values of the Li cells before and after the rate capability tests in both organic and aqueous electrolytes are also displayed in *SI Appendix*, Fig. S11 and *SI Appendix*, Tables S1 and S2. When the mass loading of Li_1.05_Mn_1.95_O_4_ to Li*_x_*Nb_2/7_Mo_3/7_O_2_ is set to 1.5, a clear increase in impedance is noted on electrochemical cycles with 21 m LiTFSA/water electrolyte. Moreover, the increase in impedance is evidenced only for Li*_x_*Nb_2/7_Mo_3/7_O_2_ and not for Li_1.05_Mn_1.95_O_4_ from the evaluation of impedance using symmetric cells (28) as clearly shown in *SI Appendix*, Fig. S11. This fact suggests that the increase in impedance originates from the surface layer formation associated with the decomposition of the TFSA anion on the surface of Li*_x_*Nb_2/7_Mo_3/7_O_2_ as described in a later section. However, better stability of impedance is also noted for the aqueous electrolyte, which is consistent with the fact that better long-term stability is realized for the aqueous electrolyte as compared in Fig. 2*C* and *SI Appendix*, Fig. S8.

**Fig. 3.**
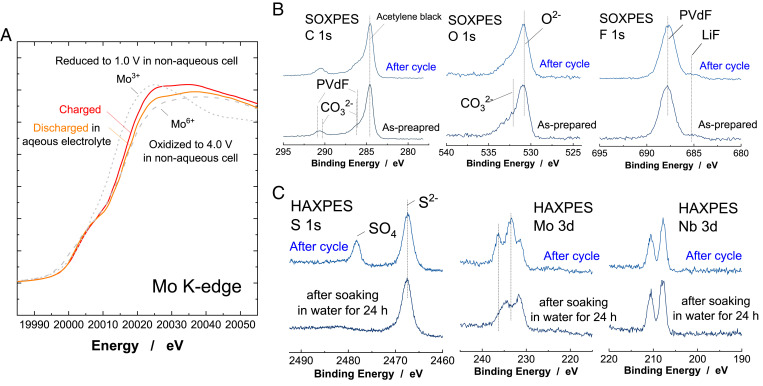
Characterization of Li*_x_*Nb_2/7_Mo_3/7_O_2_ cycled in the aqueous electrolyte. (*A*) Changes in Mo K-edge XAS spectra of Li*_x_*Nb_2/7_Mo_3/7_O_2_ after cycle in the aqueous electrolyte (the mass loading ratio of the positive electrode to negative electrode was set to 1.0). The data collected in nonaqueous electrolyte is also shown for comparison. (*B*) SOXPES spectra of the composite Li*_x_*Nb_2/7_Mo_3/7_O_2_ electrodes before and after cycle in 21 m LiTFSA/H_2_O. The full cell was cycled in the range of 0 to 2.6 V for five cycles at a rate of 10 mA ⋅ g^−1^ (the mass loading ratio; 1.5), and then the negative electrode was taken out from the cell for the measurement. (*C*) HAXPES spectra of the cycled electrode after rinse by water for a short time (denoted as “after cycle”) and after soaking in water for 24 h. Other data sets are found in *SI Appendix*, Fig. S12.

To further study the charge compensation mechanisms of Li*_x_*Nb_2/7_Mo_3/7_O_2_ in aqueous electrolyte, XAS has been performed at beamline BL-9C at Photon Factory in Japan. As shown in Fig. 3*A*, Mo K-edge XAS spectra show a clear shift on absorption energy on charge/discharge in the aqueous electrolyte. The change in the oxidation state of Mo is estimated to be one electron redox of Mo ions by comparing the reference materials of fully charged and discharged Li*_x_*Nb_2/7_Mo_3/7_O_2_ prepared in nonaqueous electrolyte, which is also consistent with the estimation from current passed through the electrode material in the cell. The surface layer formed on the negative electrode was further analyzed by SOXPES. SOXPES spectra of Li*_x_*Nb_2/7_Mo_3/7_O_2_ before and after the cycle test are shown in Fig. 3*B* and *SI Appendix*, Fig. S12. To remove TFSA ions from the cycled composite electrode, the electrode was rinsed by water for a short time and then dried in a nitrogen-filled glove box. For both electrodes, major components are assigned to originate from poly(vinylidene fluoride) binder and acetylene black (AB) used for the preparation of composite electrodes with Li*_x_*Nb_2/7_Mo_3/7_O_2_. No clear difference before or after the cycle test is noted. Also, after the cycling test, the presence of Nb and Mo near the surface is clearly evidenced, even by SOXPES (Fig. 3*B*), which suggests that the thickness of the surface layer is relatively thin, and this observation is clearly different from the case of Mo_6_S_8_. Thick (>10 nm) SEI layers are formed, and the presence of Mo and S has not been found near the surface by photoelectron spectroscopy (PES) after cycling in 21 m LiTFSA aqueous electrolyte (16). Mo_6_S_8_ has been covered with LiF as a major component of the passivation layer. Note that our study also suggests that LiF is formed (16, 18), but major components in the passivation layer seems to be derived from the decomposition of the TFSA anion, and this finding is a good agreement in literature with highly oriented pyrolytic graphite (29). Recent publication data also suggests that the SEI layer formed in 21 m LiTFSA aqueous electrolyte is easily changed from the original structure by the contact of solvents (29). Therefore, we have utilized hard X-ray photoelectron spectroscopy (HAXPES), by which the data are obtained from thicker regions (∼10 nm for HAXPES) compared with SOXPES (<2 nm). Fig. 3*C* shows HAXPES spectra for the Li*_x_*Nb_2/7_Mo_3/7_O_2_ negative electrode after the cycle test. Here, the composite electrode with Li*_x_*Nb_2/7_Mo_3/7_O_2_ was cycled in 21 m LiTFSA aqueous electrolyte and examined by HAXPES after washing with pure water for a short time. Two components at 2,478 and 2,468 eV in S 1s spectra are clearly observed, which can be assigned to SO_4_^2−^ and S^2−^ ions (30), which are derived from the decomposition reaction of TFSA anions. The peak at 2,468 eV is also probably assigned into thiol species (−SH) (29). Note that a residue of TFSA anions in electrolyte is not found because a peak from the CF_3_ functional group in the TFSA anion is not found in C 1s spectra at 293 eV (29). The SEI layer formation in 21 m LiTFSA aqueous electrolyte is triggered by the formation of hydroxide species and nucleophilic chemical reactions with TFSA anions (31). Stability of these surface layers to water has been also examined, and the cycled electrodes were soaked in water for 24 h. The peak of SO_4_^2−^ has disappeared after soaking in water for 24 h, indicating dissolution into water, whereas the peak of sulfide (or thiol) has remained. SO_4_^2−^ is not soluble in 21 m LiTFSA aqueous electrolyte but is soluble in pure water. Sulfide (or thiol) species formed on Li*_x_*Nb_2/7_Mo_3/7_O_2_ are not soluble even in pure water. Note that, in Mo 3-d HAXPES spectra, peaks at 233 and 237 eV disappear after 24 h soaking, indicating that the surface layer also contains water-soluble Mo species. This fact indicates that Mo ions are also attacked by the hydroxide species and, potentially, decomposition products of TFSA anions, resulting in the incorporation of the Mo species in the surface layer. In contrast, Nb ions seem to be chemically stable against nucleophilic chemical reactions, and no change is found after soaking in water for 24 h. From these results, it is proposed that the synergy between stable sulfide layer formation and chemically stable Nb ions results in higher durability for Li*_x_*Nb_2/7_Mo_3/7_O_2_ in the concentrated aqueous electrolyte with TFSA anions.

In summary, Li*_x_*Nb_2/7_Mo_3/7_O_2_ is studied as the large-capacity negative electrode material for aqueous LIBs. Li-excess metastable phase, Li_x_Nb_2/7_Mo_3/7_O_2_, was utilized as a negative electrode after simple oxidation by soaking in water, showing a high capacity and long cycle life for the aqueous system. HAXPES study of Li*_x_*Nb_2/7_Mo_3/7_O_2_ reveals the presence of protective passivation layers at the negative electrode after cycling in 21 m LiTFSA. Effective suppression of the hydrogen evolution reaction was achieved by the surface layer formed by the sacrificial decomposition of electrolyte used, which significantly enhanced the available capacity of Li*_x_*Nb_2/7_Mo_3/7_O_2_. The optimized aqueous Li cells with Li_1.05_Mn_1.95_O_4_ and Li*_x_*Nb_2/7_Mo_3/7_O_2_ deliver high energy density of 107 Wh ⋅ kg^−1^, even at a slow rate of 10 mA ⋅ g^−1^, and high durability (∼73% of capacity retention over 2,000 cycles at 100 mA ⋅ g^−1^) is realized. It should be emphasized that there is still room for even larger capacity because approximately half of the available capacity of Li*_x_*Nb_2/7_Mo_3/7_O_2_ (340 mA ⋅ h ⋅ g^−1^ as shown in Fig. 1*A*), which was determined in organic media, could be utilized in the aqueous electrolyte. Recently, reversible Li-Al alloy formation (operating voltage; <1 V versus Li) is achieved in the aqueous electrolyte (32). The full capacity of Li*_x_*Nb_2/7_Mo_3/7_O_2_ is possibly utilized as the negative electrode material in this voltage range. We believe that our work will nicely contribute to the development of high-energy aqueous LIBs by providing a class of high-capacity negative electrode materials.

## Materials and Methods

### Material Synthesis.

Li_9/7_Nb_2/7_Mo_3/7_O_2_ was prepared from a mixture of LiMoO_2_ and Li_3_NbO_4_ by mechanical milling at 600 rpm with a zirconia container and balls (22). Li_3_NbO_4_ was prepared from Li_2_CO_3_ (98.5%; Kanto Kagaku) and Nb_2_O_5_ (99.9%; Wako Pure Chemical Industries) at 950 °C for 24 h in air. LiMoO_2_ was synthesized from Li_2_CO_3_ and MoO_3_ (99.5%; Kanto Kagaku) with AB (HS-100; Denka) at 800 °C in Ar. Li_1.05_Mn_1.95_O_4_ was prepared from Li_2_CO_3_ and MnCO_3_ (Kishida Chemical) at 750 °C for 24 h in air.

### Electrochemical Characterization.

Li*_x_*Nb_2/7_Mo_3/7_O_2_ was mixed with 10 wt% AB (HS-100; Denka) by ball milling at 300 rpm. Composite electrodes consisted of 76.5 wt% Li*_x_*Nb_2/7_Mo_3/7_O_2_, 13.5 wt% AB, and 10 wt% poly(vinylidene fluoride) pasted on aluminum foil as a current collector. Two-electrode cells (TJ-AC; Tomcell Japan) were assembled in the Ar-filled glove box. A glass filter (GB-100R, Advantec) was used as separator. LiTFSA was purchased from Wako Pure Chemical Industries and used as received.

### Surface Analysis by Photoelectron Spectroscopy.

For SOXPES measurement, Quantera SXM (ULVAC-PHI) equipped with an Al-Kα X-ray source (1486.6 eV) was utilized. HAXPES measurement was carried out using a custom-made MULTIPROBE system (Omicron Nanotechnology) equipped with Cr Kα (5414.9 eV) focused X-ray source (Ulvac-Phi) and EW-4000 electron analyzer (VG Scienta). The incident angle of X-rays and takeoff angle of photoelectrons, as measured from the sample surface, were fixed at 75° and 90°, respectively. This system was directly attached to an Ar-filled glove box so that the electrochemically treated composite electrodes could be directly transferred to the analysis chamber without exposing them to open air.

## Supplementary Material

Supplementary File

## Data Availability

All data related to the study are included in the article and supporting information. The raw data supporting in Figs. 1–3, respectively, are available for public access at Mendeley Data (https://doi.org/10.17632/9t78p6h9hc.1) (37).
