# Supplementary material for: Nanosized and metastable molybdenum oxides as negative electrode materials for durable high-energy aqueous Li-ion batteries
Source: Proc Natl Acad Sci U S A. 2021 Nov 23;118(48):e2024969118. doi: 10.1073/pnas.2024969118 (PMC8640940; doi:10.1073/pnas.2024969118)
Supplement: Supplementary File [file pnas.2024969118.sapp.pdf]

Supporting Information for

Nanosized and Metastable Molybdenum Oxides as Negative Electrode Materials for Durable High-energy Aqueous Li-ion Batteries

Jeongsik Yun[a,b], Ryota Sagehashi[c], Yoshihiko Sato,[d] Takuya Masuda,[e] Satoshi Hoshino,[c] Hongahally Basappa Rajendra,[d] Kazuki Okuno[f], Akihisa Hosoe[f] Aliaksandr S. Bandarenka[a,b], and Naoaki Yabuuchi[d, g, h*]

a - Physics of Energy Conversion and Storage, Physik-Department, Technische Universität München, James-Franck-Str. 1, 85748 Garching, Germany

b – E-Conversion, Schellingstraße 4, 80799 Munich, Germany

c – Department of Applied Chemistry, Tokyo Denki University, 5 Senju Asahi-Cho, Adachi, Tokyo 120-8551, Japan

d – Department of Chemistry and Life Science, Yokohama National University, 79-5 Tokiwadai, Hodogaya-ku, Yokohama, Kanagawa 240-8501, Japan

e – Research Center for Advanced Measurement and Characterization, National Institute for Materials Science (NIMS), 1-1 Namiki, Tsukuba, Ibaraki 305-0044, Japan

f – Energy and Electronics Materials R&D Laboratories, Sumitomo Electric Industries, Ltd., 1-1-3, Shimaya, Konohana-ku, Osaka 554-0024, Japan

g –Advanced Chemical Energy Research Center, Yokohama National University, 79-5 Tokiwadai, Hodogaya-ku, Yokohama, Kanagawa 240-8501, Japan

h – Elements Strategy Initiative for Catalysts and Batteries, Kyoto University, Kyoto, f1-30 Goryo-Ohara, Nishikyo-ku, Kyoto 615-8245, Japan

*Corresponding Author

E-mail: yabuuchi-naoaki-pw@ynu.ac.jp

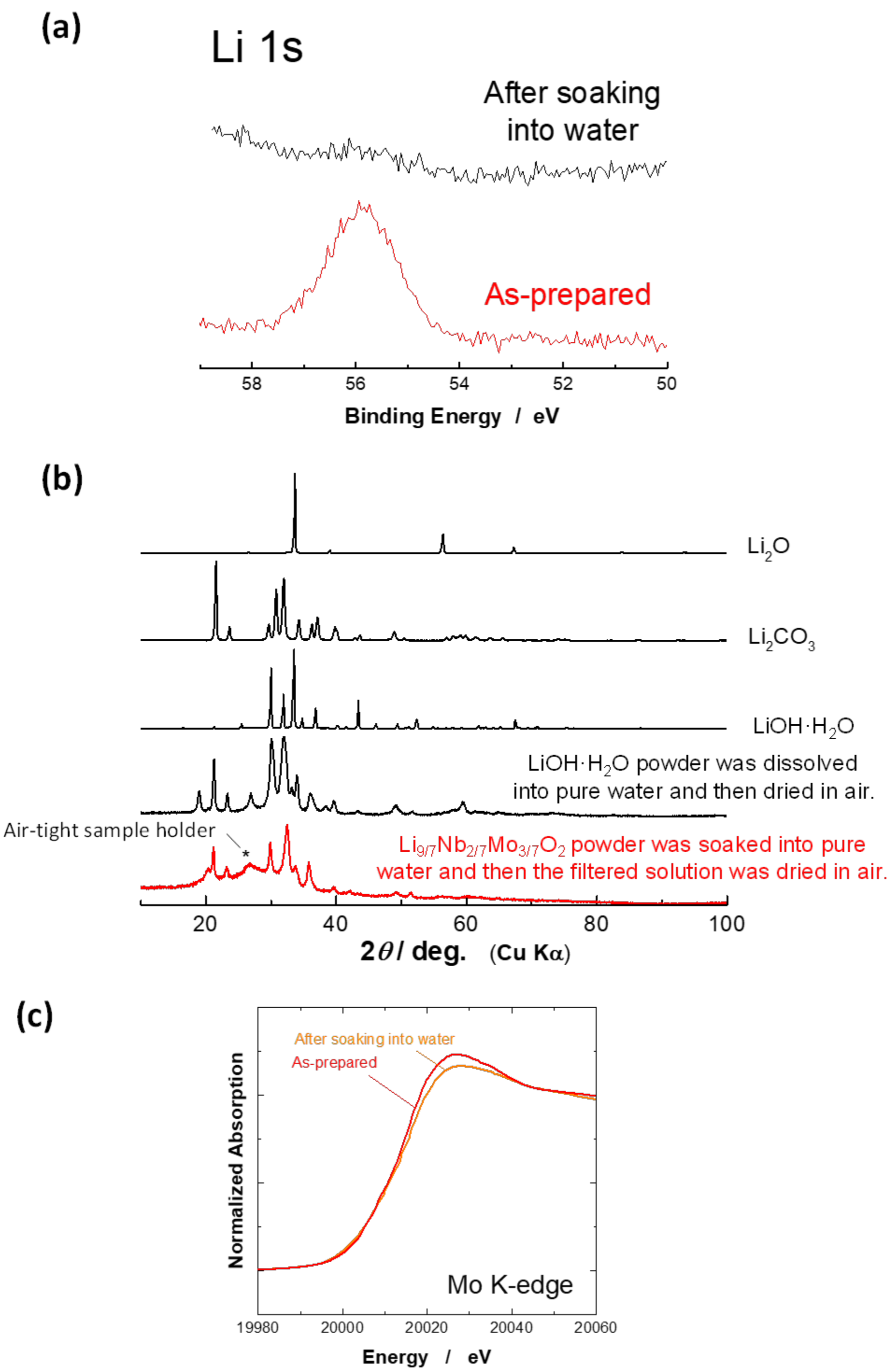

Figure S1. (a) SOXPES spectra of Li 1s core peaks of $Li_xNb_{2/7}Mo_{3/7}O_2$ before and after soaking into water. (b) An XRD pattern of the powder obtained by drying the aqueous solution, in which $Li_{9/7}Nb_{2/7}Mo_{3/7}O_2$ was soaked. The value of pH of the aqueous solution was 12.1 after soaking of

$Li_{9/7}Nb_{2/7}Mo_{3/7}O_2$. For comparison, $LiOH \cdot H_2O$ was dissolved into pure water, and then dried in air. Similar XRD patterns are obtained for both dried powders. XRD patterns of Li_2O, Li_2CO_3, and $LiOH \cdot H_2O$ are also shown as reference materials. The dried powders seem to be a mixture of Li_2CO_3, and $LiOH \cdot H_2O$. (c) X-ray absorption spectra at Mo K-edge of $Li_{9/7}Nb_{2/7}Mo_{3/7}O_2$ before and after soaking water. A clear shift of energy to a higher energy region indicates the oxidation of Mo ions and Li extraction by water soaking.

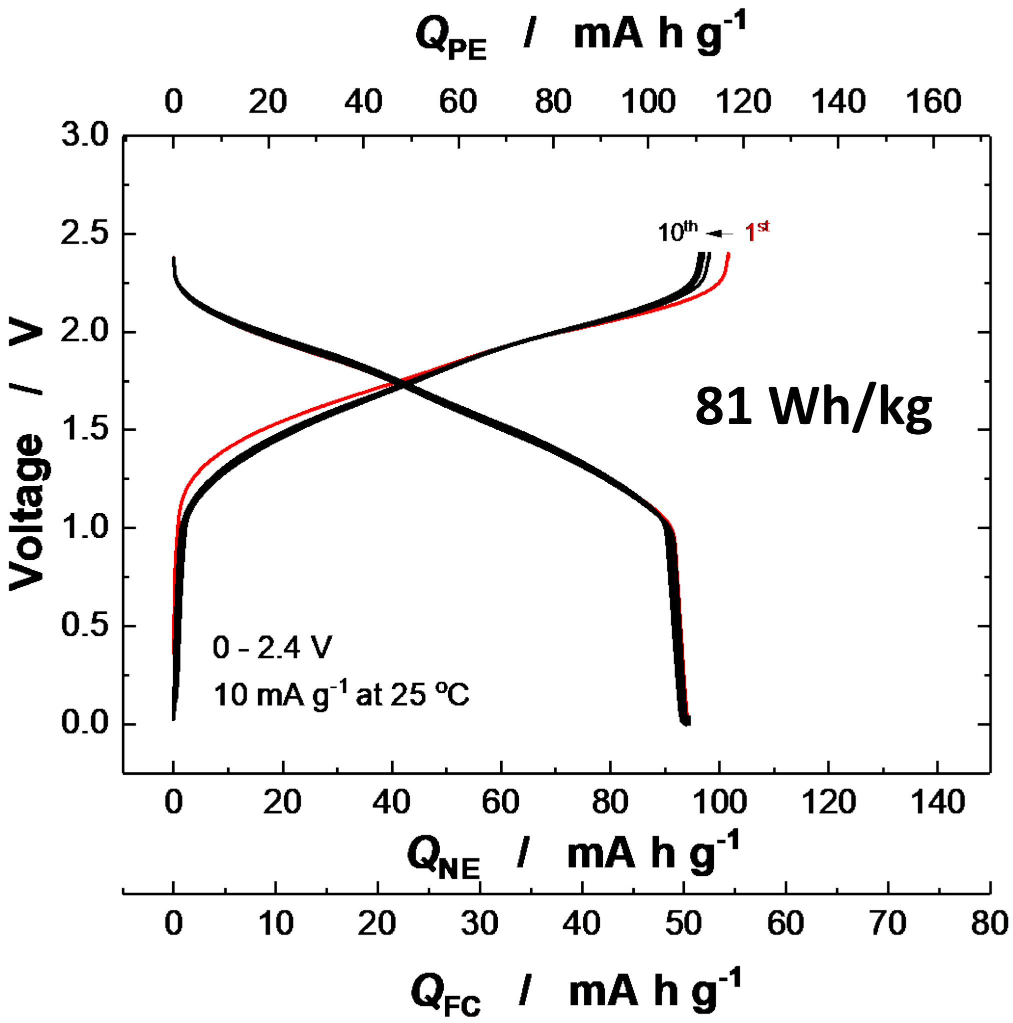

Figure S2. Charge/discharge curves of $Li_{1.05}Mn_{1.95}O_4$/$Li_xNb_{2/7}Mo_{3/7}O_2$ (w/w = 0.86:1.0) full cell at a rate of 10 mA g^{-1} in 21 m LiTFSA/H_2O.

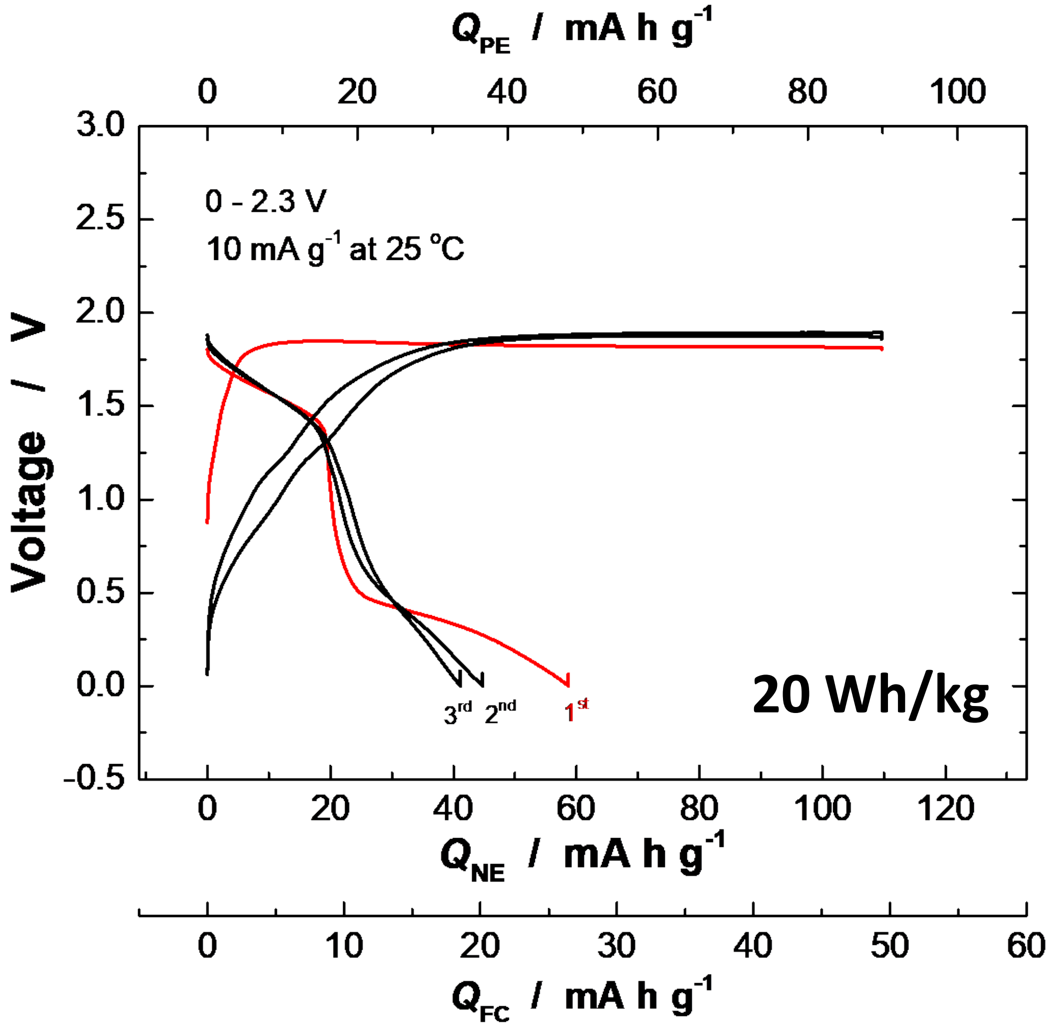

Figure S3. Charge/discharge curves of $Li_{1.05}Mn_{1.95}O_4/Li_xNb_{2/7}Mo_{3/7}O_2$ (w/w = 0.82:1.0) full cell at a rate of 10 mA g^{-1} in 1 M LiTFSA/H_2O.

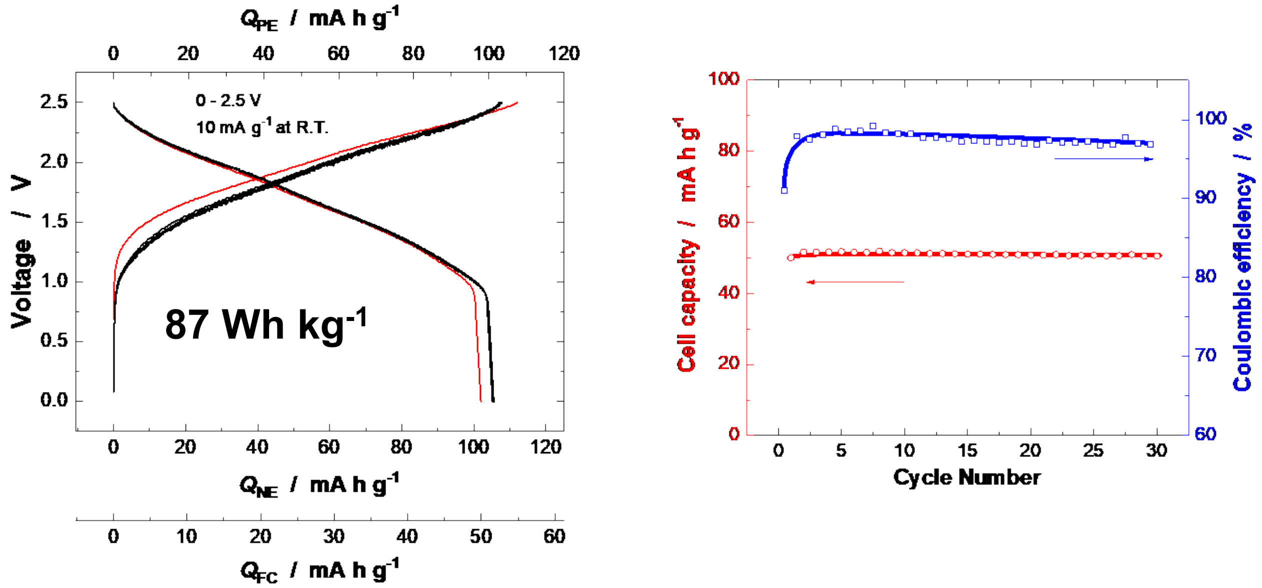

Figure S4. Charge/discharge curves of $Li_{1.05}Mn_{1.95}O_4$/$Li_xNb_{2/7}Mo_{3/7}O_2$ (w/w = 0.86:1.0) full cell at a rate of 10 mA g^{-1} in 1 M $LiPF_6$/EC:DMC. Its capacity retention and Coulombic efficiency for 30 cycles are also plotted.

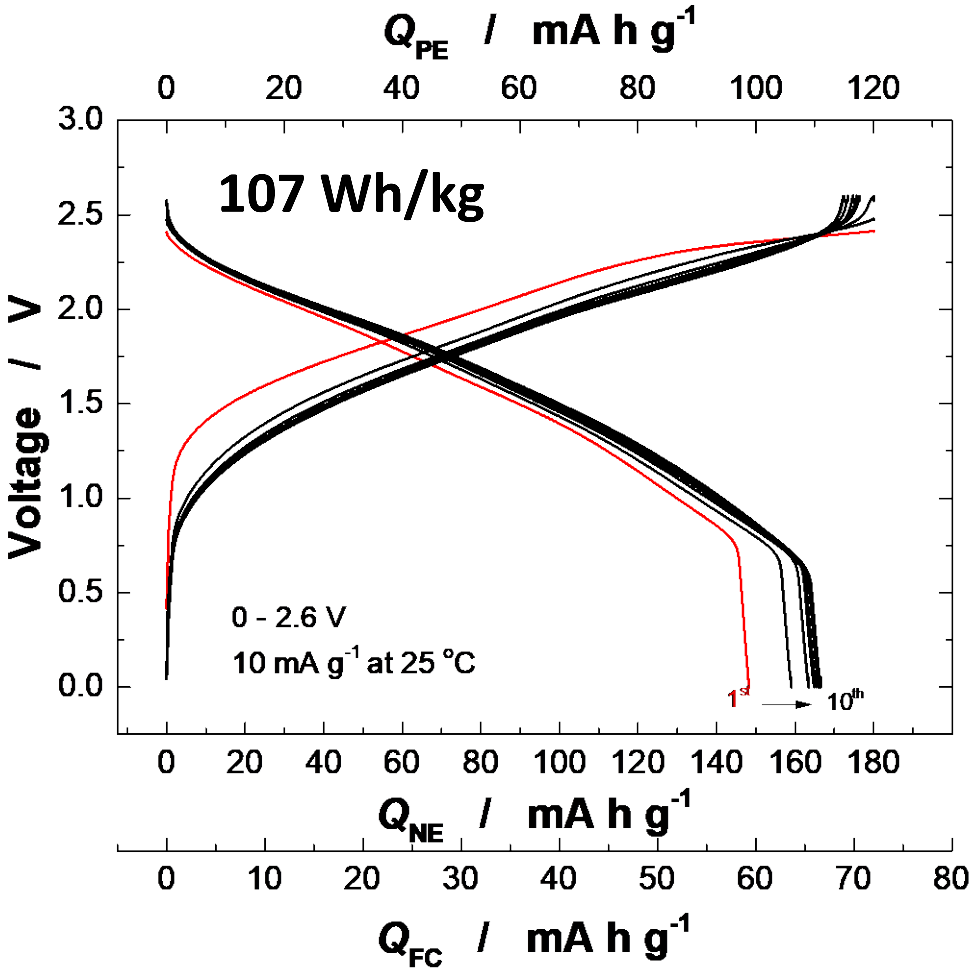

Figure S5. Charge/discharge curves of $Li_{1.05}Mn_{1.95}O_4$/$Li_xNb_{2/7}Mo_{3/7}O_2$ (w/w = 1.5:1.0) full cell at a rate of 10 mA g^{-1} in 21 m LiTFSA/H_2O.

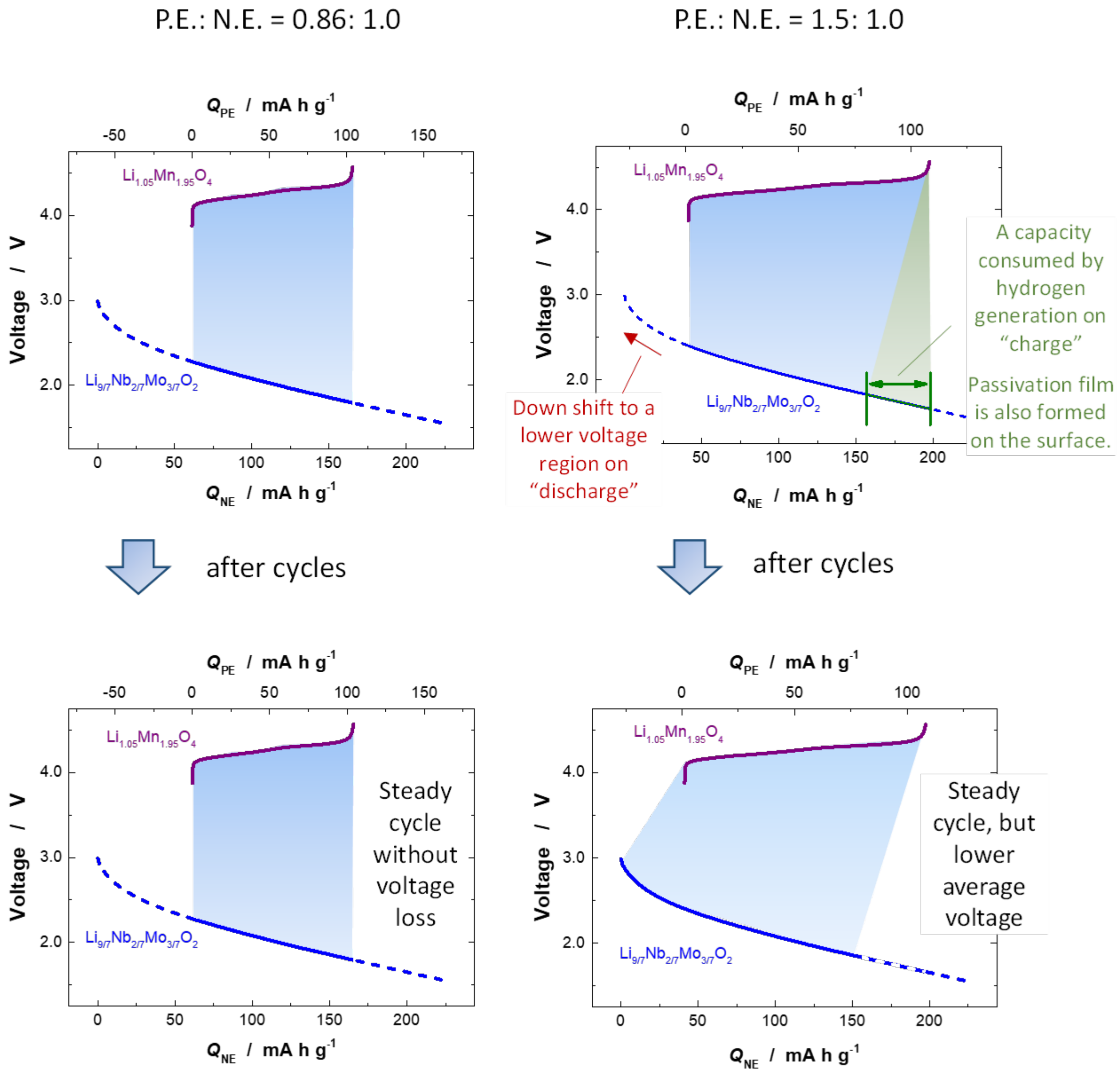

Figure S6. Schematic illustrations of changes in voltage profiles of full cells with different ratios of positive/negative electrode materials associated with imbalanced Coulombic efficiency for both electrodes.

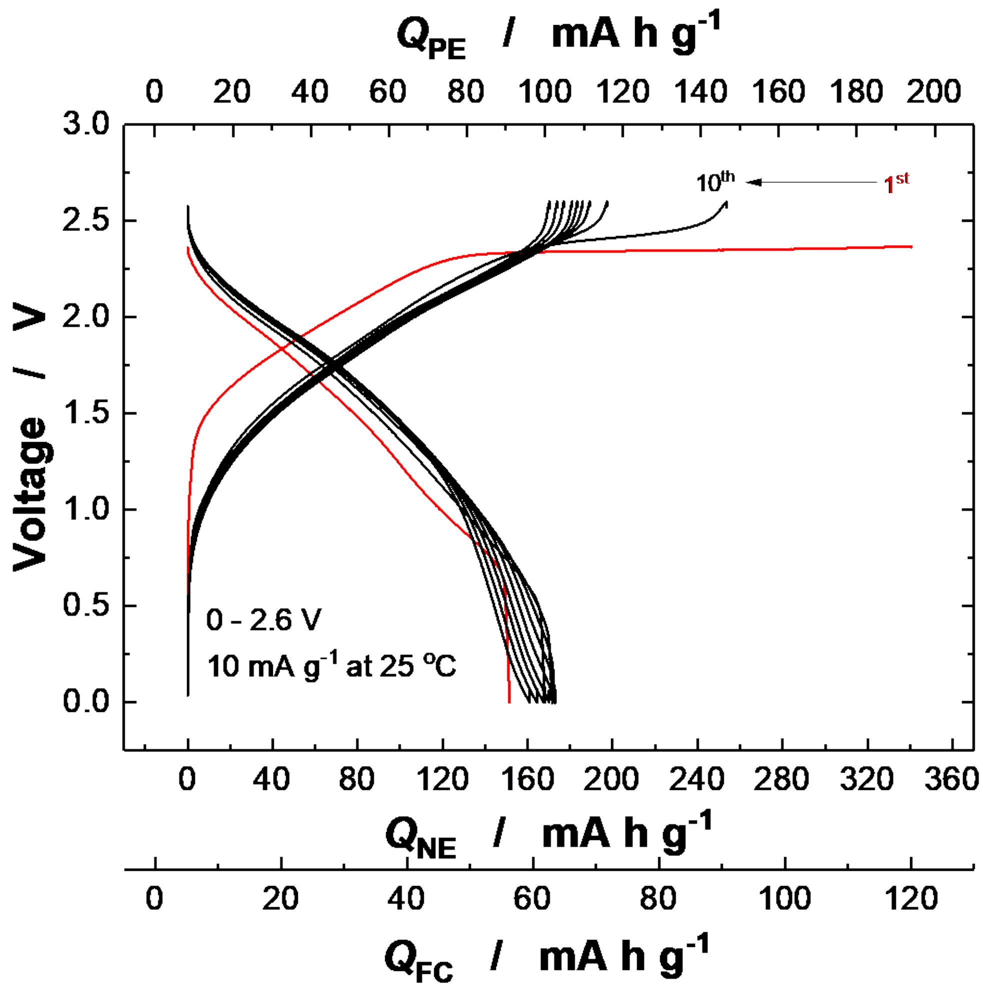

Figure S7. Charge/discharge curves of $Li_{1.05}Mn_{1.95}O_4$/$Li_xNb_{2/7}Mo_{3/7}O_2$ (w/w =1.7:1.0) full cell at a rate of 10 mA g^{-1} in 21 m LiTFSA/H_2O.

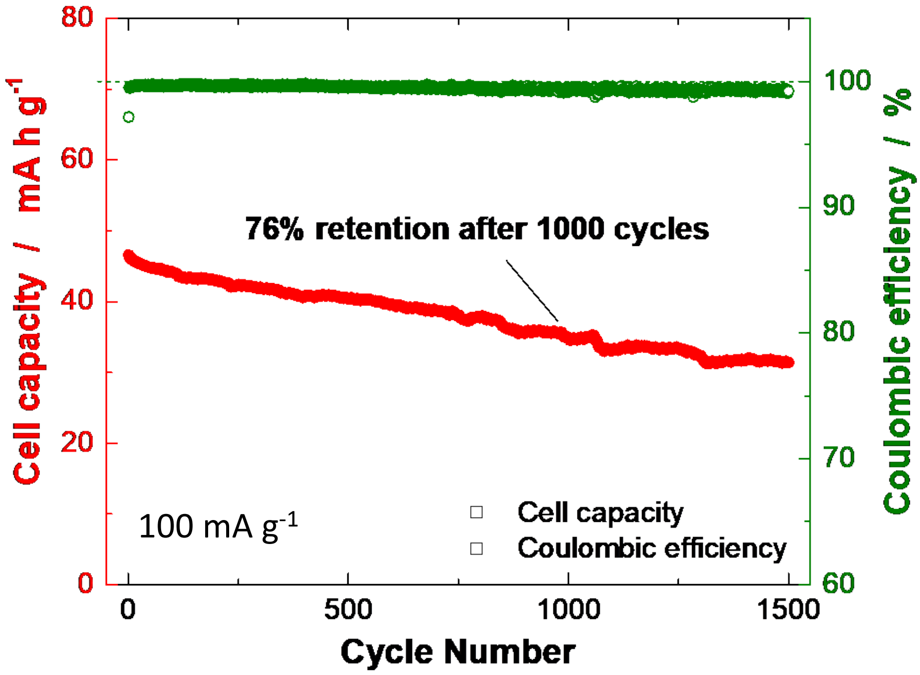

Figure S8. Long-term cycling stability performance of the full cell for 1500 cycles at a rate of 100 mA g^{-1} in 1 M $LiPF_6$/EC:DMC.

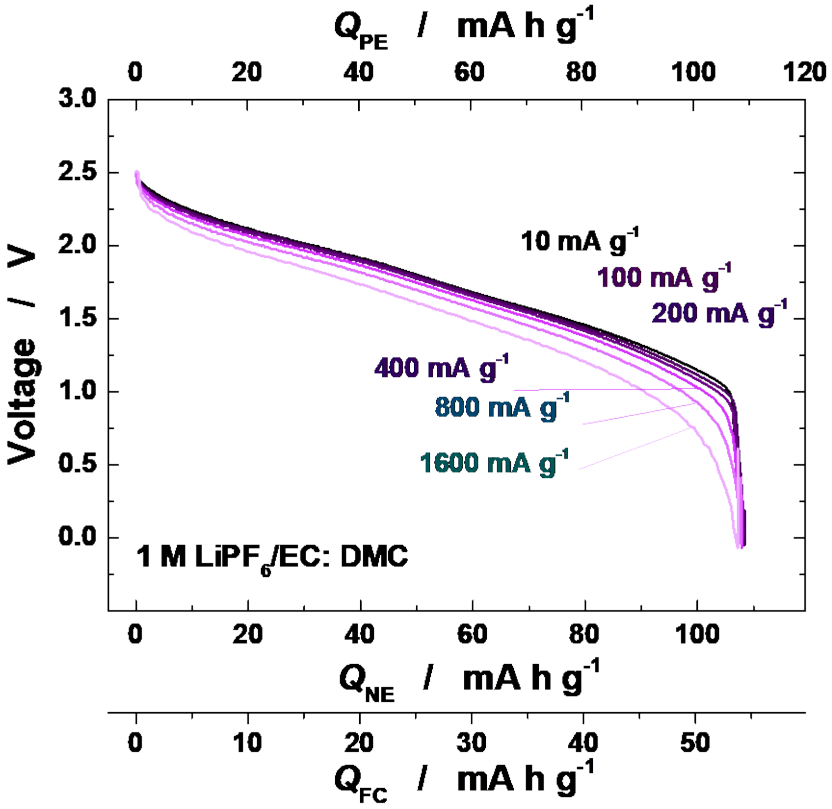

Figure S9. Rate capability of the full cell in 1 M $LiPF_6$/EC:DMC.

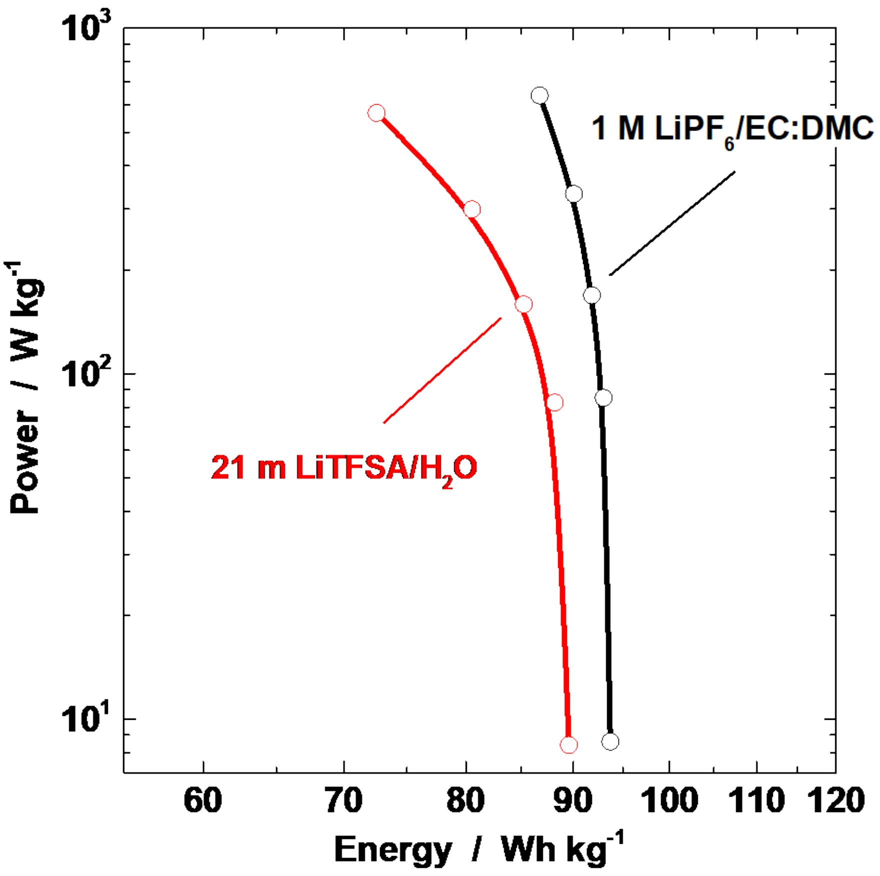

Figure S10. A Ragone plot for $Li_{1.05}Mn_{1.95}O_4/Li_xNb_{2/7}Mo_{3/7}O_2$ full cells in different electrolyte solutions.

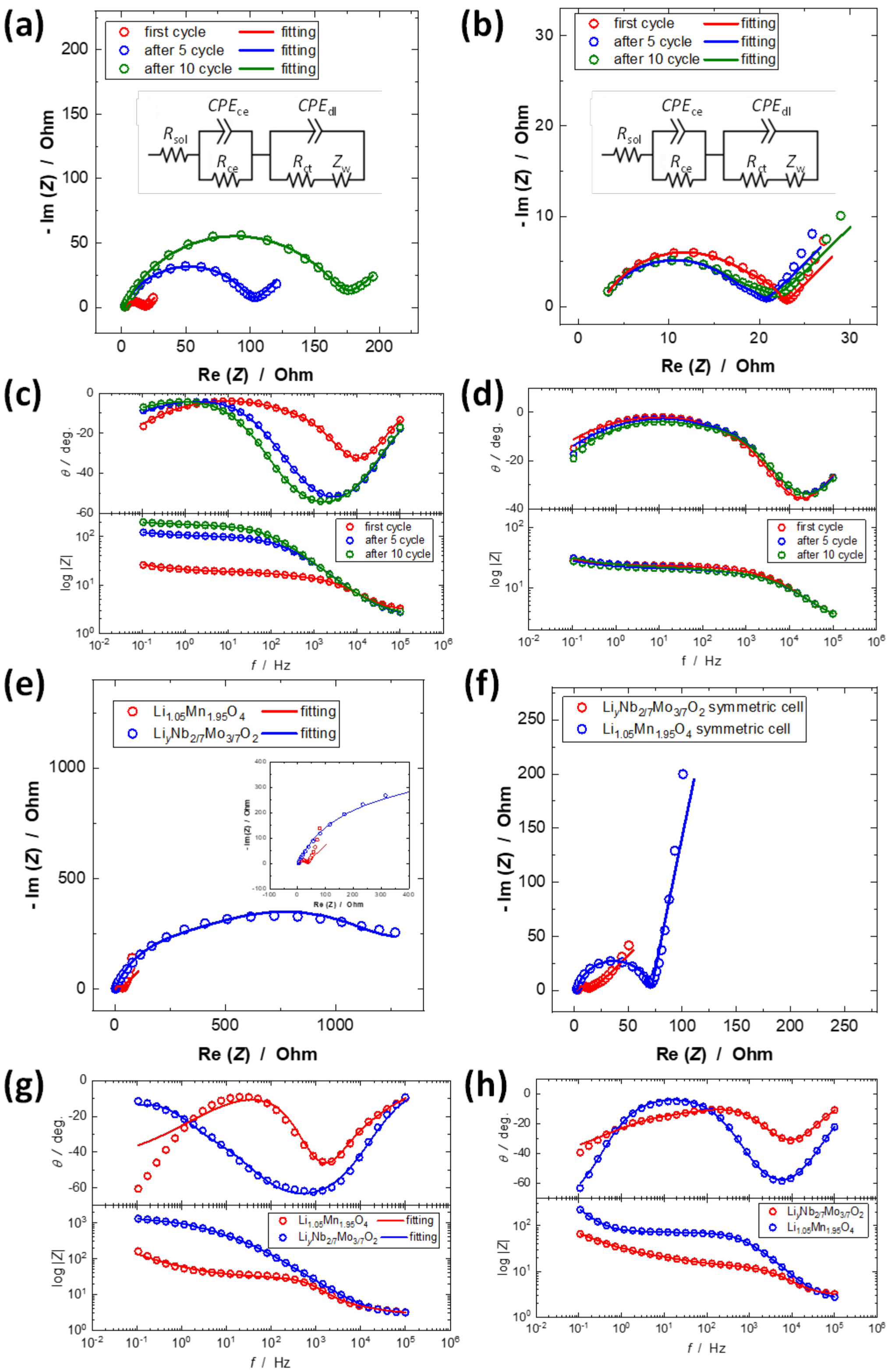

Figure S11. (a-d) Observed impedance data and corresponding fitted data of $Li_{1.05}Mn_{1.95}O_4$ /$Li_xNb_{2/7}Mo_{3/7}O_2$ full cells (w/w =1.5:1.0) at 50% state-of-charge that were cycled in (a, c) 21 m

LiTFSA/water and (b, d) 1 M $LiPF_6$ in EC:DMC (3:7) for the first, fifth and tenth cycles at 10 mA g^{-1}. Utilized equivalent electric circuit models are shown in the inset figure. Herein, R_{sol} indicates the resistance of electrolyte solutions. R_{ce} corresponds to the resistance in the composite electrodes (electron transfer from current collector to all active material powder, including the contact resistance for active material powders and conductive carbon). R_{ct} indicates the charge transfer resistance of Mn/Mo redox reaction coupled with Li insertion/extraction. Two constant phase elements (CPE) are also utilized for capacitance components. Fitted data is also summarized in **Supporting Table S1**. Increases in impedance are clearly observed for 21 m LiTFSA/water electrolyte, suggesting that surface film formation on electrochemical cycles.

Two sets of full cells (w/w =1.5:1.0) with aqueous and non-aqueous electrolyte solutions were cycled for 10 cycles at 10 mA g^{-1}, and then electrodes were taken out from the cells after charge (50% state-of-charge). By using these electrodes, four symmetric cells were assembled; $Li_{1.05}Mn_{1.95}O_4$/$Li_{1.05}Mn_{1.95}O_4$ and $Li_xNb_{2/7}Mo_{3/7}O_2$/$Li_xNb_{2/7}Mo_{3/7}O_2$ symmetric cells with aqueous and non-aqueous electrolyte solutions. (e-h) Observed impedance data and corresponding fitted data of the $Li_{1.05}Mn_{1.95}O_4$/$Li_{1.05}Mn_{1.95}O_4$ and $Li_xNb_{2/7}Mo_{3/7}O_2$/$Li_xNb_{2/7}Mo_{3/7}O_2$ symmetric cells at 50% state-of-charge in (e, g) 21 m LiTFSA/water and (f, h) 1 M $LiPF_6$ in EC:DMC (3:7). Fitted data is also shown in **Supporting Table S2**. The data indicates that $Li_xNb_{2/7}Mo_{3/7}O_2$ is responsible for the increase in impedance with aqueous electrolyte.

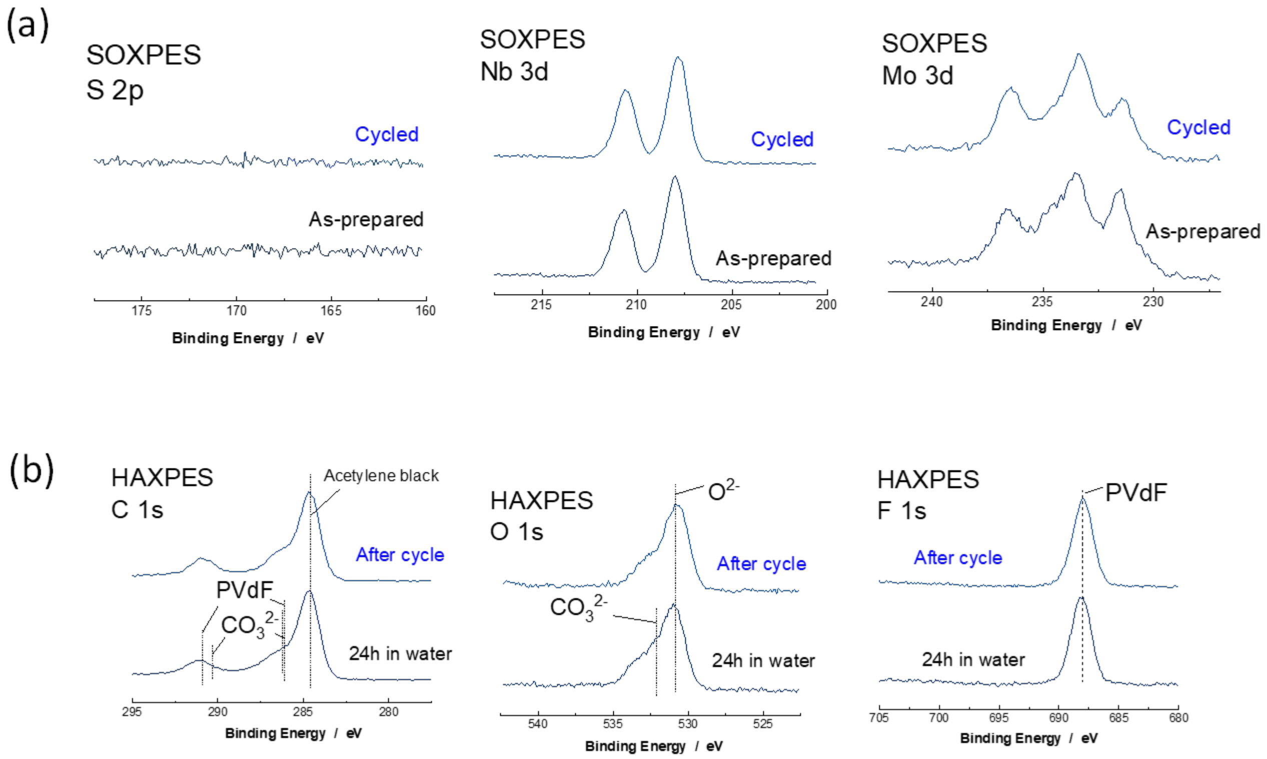

Figure S12. (a) SOXPES spectra of the composite $Li_xNb_{2/7}Mo_{3/7}O_2$ electrodes before and after cycle in 21 m LiTFSA/H_2O. The full cell was cycled in the range of 0 to 2.6 V for 5 cycles at a rate of 10 mA g^{-1} (the mass loading ratio; 1.5), and then the negative electrode was taken out from the cell for the measurement. (b) HAXPES spectra of the cycled electrodes after rinse by water and after soaking in water for 24 h.

Table S1. Fitting results of the EIS spectra from the full cells in 21 m LiTFSA/water and 1 M $LiPF_6$ in EC/DMC (3:7), respectively. Each full cell consists of $Li_yNb_{2/7}Mo_{3/7}O_2$ and $Li_{1.05}Mn_{1.95}O_4$.

Aqueous	*R_Sol*	*R_CL*	*R_CT*	*Z_W*	*Q_CL*	*n*	*Q_DL*	*n*
1st	*2.88*	*7.66*	*8.10*	*5.67*	*0.00115*	*0.590*	*1.42E-05*	*0.881*
6th	*2.26*	*72.6*	*25.6*	*156.0*	*8.13E-05*	*0.748*	*9.71E-05*	*0.758*
11st	*2.29*	*168.6*	*29.6*	*18.9*	*8.84E+06*	*0.735*	*0.0358*	*0.487*
Organic	*R_Sol*	*R_CL*	*R_CT*	*Z_W*	*Q_CL*	*n*	*Q_DL*	*n*
1st	*2.42*	*1.76*	*18.3*	*4.52*	*0.000420*	*1.00*	*2.78E-05*	*0.736*
6th	*2.21*	*1.45*	*16.5*	*5.38*	*0.00153*	*0.928*	*3.86E-05*	*0.705*
11st	*2.17*	*2.72*	*16.4*	*7.20*	*0.00286*	*0.749*	*3.83E-05*	*0.704*

Table S2. Fitting results of the EIS spectra from the symmetric cells in 21 m LiTFSA/water and 1 M $LiPF_6$ in EC/DMC (3:7), respectively. Each symmetric cell consists of either two $Li_yNb_{2/7}Mo_{3/7}O_2$ or two $Li_{1.05}Mn_{1.95}O_4$ electrodes that were obtained from the cycled cells.

Aqueous	*R_Sol*	*R_CL*	*R_CT*	*Z_W*	*Q_CL*	*n*	*Q_DL*	*n*
LMO	*2.82*	*2.55*	*23.4*	*63.5*	*0.000207*	*0.678*	*1.08E-05*	*1.00*
LiNbMoO	*2.80*	*741.2*	*308.3*	*186.3*	*0.000182*	*0.771*	*5.90E-05*	*0.790*
Organic	*R_Sol*	*R_CL*	*R_CT*	*Z_W*	*Q_CL*	*n*	*Q_DL*	*n*
LMO	*2.26*	*69.3*	*40.9*	*4010*	*1.07E-05*	*0.852*	*0.00696*	*0.883*
LiNbMoO	*2.95*	*5.91*	*9.54*	*30.1*	*0.00858*	*0.609*	*1.94E-05*	*0.848*
